# Effect of Helmert Transformation Parameters and Weight Matrix on Seasonal Signals in GNSS Coordinate Time Series

**DOI:** 10.3390/s18072127

**Published:** 2018-07-03

**Authors:** Guo Chen, Qile Zhao, Na Wei, Min Li

**Affiliations:** 1 School of Geodesy and Geomatics, Wuhan University, No. 129 Luoyu Road, Wuhan 430079, China; guo_chen@whu.edu.cn; 2 GNSS Research Center, Wuhan University, No. 129 Luoyu Road, Wuhan 430079, China; lim@whu.edu.cn; 3 Collaborative Innovation Center of Geospatial Technology, Wuhan University, No. 129 Luoy Road, Wuhan 430079, China

**Keywords:** seasonal signals, environmental loading deformation, position series stacking, WRMS reduction, velocity bias

## Abstract

Seasonal signals caused by the Earth’s surface mass redistribution can be detected by Global Navigation Satellite Systems (GNSS). The authors analyze the effect of Helmert transformation parameters and weight matrices, as well as the additional draconic signals on seasonal signals, in the GNSS coordinate time series. Moreover, the contribution of environmental loading models to the GNSS position series is assessed. Position time series of 647 global stations, with spans of 2–21 years are collected to generate six cumulative solutions using different parameters estimated in a deterministic model, as well as weight matrices. Comparison among the different solutions indicates that Helmert transformation parameters and weight matrices can result in a root mean square of 0.1 mm and 0.3 mm for seasonal signals, respectively. Compared to the displacements obtained from environmental loading models, seasonal signals estimated with the Helmert parameters and full weight matrices considered seems to have the best agreement with the results of the loading model. Meanwhile, the additional draconic signals are not effective to be parameterized in the deterministic model with an observation time span less than 15 years, marginally. There are 62%, 72% and 90% of 647 stations with weight root mean squares (WRMS) reduced by removing the loading-model-induced changes from the GNSS residual series for the east, north and vertical components, respectively. Finally, to obtain a velocity estimation with a bias of less than 0.1 mm/yr induced by seasonal signals, the position series with a time span greater than seven years is suggested.

## 1. Introduction

Global Navigation Satellite System (GNSS) has been widely applied in different fields and have been improved recently. Since there was a lack of common standards on the data analyses and products service, the International GNSS Service (IGS) was established and has been providing global available official products related to GNSS, especially the Global Positioning System (GPS), since 1994 [1].

Many efforts have been expended on geophysical explanations and systematic errors with the help of high-quality GNSS products. The strong periodic signals are observed in the IGS core products (such as satellite orbit, station position and Earth rotation parameters), which is the result of various factors.

The geophysical factors are mainly the Earth’s surface mass redistribution, including the atmospheric pressure variation, non-tidal oceanic loading and hydrological loading. van Dam and Herring [2] found an approximate 60% change in the baseline length determined by very long baseline interferometry (VLBI), which is caused by atmospheric pressure loading. The deformations caused by atmospheric pressure variations are mainly in a vertical direction which could be ten times larger than the horizontal [3]. Compared to the solution without atmospheric pressure loading correction, the repeatability of solutions, with regression factors estimated between station displacement and the local pressure, improves by about 20% [4]. Considering the correction of non-tidal oceanic loading with a higher resolution barotropic ocean model, Boy et al. [5] found that the surface gravity measurements due to storm surges over the North-western European shelf have a significant and systematic reduction of gravity residuals. Using 17 stations around the southern North Sea, with a 3–4 year position series, Williams and Penna [6] also recommended that a higher resolution is preferred to correct the deformation caused by non-tidal ocean loading with the metric of variance reduction. van Dam et al. [7] demonstrated that 80% of total RMS reduction is related to the annual frequency in ocean bottom pressure (OBP) data and 65% of the 700 global stations with reduced root mean square (RMS) errors was reprocessed for GPS height residuals by the Massachusetts Institute of Technology (MIT). Compared to the displacements caused by atmospheric pressure and hydrological loading, Xu [8] indicated that the displacements caused by non-tidal oceanic loading is significant only in these areas near enclosed seas and along the Antarctic coastline.

Added to the displacements caused by atmospheric pressure and non-tidal oceanic loading, van Dam et al. [9] compared the vertical displacements caused by continental water loading using the selected 147 global GPS stations, with a monthly-averaged position from 1994 to 1998 and found more than half of the stations have an annual harmonic in phase and comparable amplitude. Bevis et al. [10] focused on one GPS station near the center of the Amazon Basin and found an anti-correlation between the height of the river stage and the GPS vertical displacement, which indicates that hydrological loading plays an important role in the observed oscillations of the GPS position series. Using the hydrological loading deformations derived from Gravity Recovery and Climate Experiment (GRACE), significant weight root mean square (WRMS) reductions of GPS residuals were also validated in the Amazon Basin [11] and Nepalese Himalaya [12]. Jiang et al. [13] compared the combined contributions of atmospheric pressure, non-tidal oceanic and hydrological loading with environmental loading models from different agencies. The best performance among the different models is 74% of a total 233 stations with reduced WRMS in a vertical direction for the optimum model data (OMD).

Dong et al. [14] illustrated that the deformation caused by these seasonal surface mass redistributions can contribute to less than half of the power in the GPS annual vertical position variations. The remaining contributions might relate to unmodeled tropospheric effects, tide loading, bedrock thermal expansion, errors in phase center variation and orbit dynamic models. Regarding the technique related factors, anomalous signals (such as GPS draconitic errors with 1.04 ± 0.008 cycle per year (cpy) base frequency) were found in the spectra of the GPS position series [15], which is the result of satellite consternation repeating its inertial orientation with respect to the Sun. Meanwhile, the same anomalous signals are not validated in the Satellite Laser Ranging (SLR) or Very Long Baseline Interferometry (VLBI) solutions, or in the environmental loading displacement series. Amiri–Simkooei [16] detected a significant signal with a period of 351.6 ± 0.2 days and its higher harmonics in the GPS daily solutions from the Jet Propulsion Laboratory (JPL). The least square harmonic estimation (LS–HE) was used in his study and the variations of these periodic signals are about ±3 mm, ±3.2 mm and ±6.6 mm for the north, east and vertical components, respectively. Griffiths and Ray [17] suggested the anomalous signals in IGS products are the results of the mismodeling of orbit dynamics and the aliasing of near sidereal local station multipath effects. Concerning the orbit dynamics, Rodriguez–Solano et al. reduced the draconic errors in the GNSS geodetic products using an adjustable box-wing model rather than an empirical 5-parameter solar radiation pressure (SRP) model proposed by the Center for Orbit Determination in Europe (CODE) [18,19]. Compared to the CODE 5-parameter SRP model, the average reductions of 41%, 39% and 35% for the power spectrum of the station position were obtained for the north, east and vertical, respectively [20]. Regarding the aliasing signals, Penna et al. [21] also explained how the unmodeled and/or residual crustal tides could induce aliased periodic signals into annual and semi-annual periodic signatures for the height component. Xu et al. [22] investigated the surface displacements due to land temperature variation and the results of numerical simulation showed that the maximum displacements can reach 3 mm and 1.5 mm for vertical and horizontal components, respectively.

Concerning the periodic signals in the GNSS station position series, both seasonal signals (the annual and semi-annual signals) and draconic signals are validated by previous investigations. However, most investigations extracted seasonal signals from the position time series which were aligned to the same International Terrestrial Reference Frame (ITRF). Neither the correlations among different stations were used, nor a similarity transformation between the daily/weekly solution and the inner long-term reference was established [10,11,12]. Do the weight matrix or the transformation parameters have an impact on the seasonal signals? Moreover, should researchers estimate the draconic signals simultaneously when extracting the seasonal signals using the position time series with a finite time span? Last but not less important, using the latest IGS reprocess solutions, how much does the environmental loading model contribute to the seasonal signals derived from the GNSS position series? To answer these questions, the paper is organized as follows. An introduction to the GNSS position series sources and data processing strategies is presented in Section 2. To make a comparison between the GNSS seasonal signals and external ones, the environmental loading displacements series from the Global Geophysical Fluids Center (GGFC) are introduced and used to predict seasonal signals in Section 3. The comparison of seasonal signals within different strategies in the GNSS position series stacking is shown in Section 4 and the impact of seasonal signals on velocity estimates in GNSS series stacking solution isanalyzed in Section 5. Finally, the study is discussed and concluded in Section 6.

## 2. GNSS Station Position Series Source and Processing Strategy

The IGS second reprocessed daily solutions are collected and the model for the position time series stacking is introduced. Then, cumulative solutions with different strategies for seasonal signals are listed for further comparison.

### 2.1. GNSS Station Position Time Series Source

Continuous improvement of models and methodology result in the data reprocess requested by the IGS analysis center. IGS has completed two data reprocessing campaigns so far. The second reanalysis of the full history of data since 1994 was finalized in 2015. Compared to the first reanalysis, the main characteristics are focused in the second reprocess. The IGS operational weekly coordinate solution was switched to daily solution from GPS week 1702 (19 August 2012) to avoid the loss of signal derived from atmospheric press loading (the diurnal and semi-diurnal signals) and the daily solutions also were produced in the reanalysis. Regarding the satellite during eclipse seasons, a modification for GPS Block IIA/IIR satellite orientation was suggested and new yaw-attitude models were developed and adopted for GPS Block IIF and GLONASS-M satellites [23,24]. Since a systematic bias of 1–2 cm was found in the SLR residuals for the GNSS orbit products, which is caused by the reflected and retransmitted radiation from the Earth, the recommended model developed by Rodrigueez–Solano [25] was adopted in the reanalysis. The IERS 2010 Conventions [26], in particular the conventional mean pole model, the geopotential model, tidal variations in the Earth’s rotation and station displacements and second-order ionospheric correction also were implemented (http://acc.igs.org/reprocess2.html). The antenna calibrations have a significant impact on station coordinates, which Figurski and Nykiel [27] indicated, that the switch of antenna calibrations from IGb08 to IGS14 could cause changes of station coordinates up to several millimeters. To generate high internal consistent GNSS-related products for the second reanalysis, the unified calibration of satellites and GNSS receiver antennas (igs08.atx) was adopted by different analysis centers. As a result, the daily solutions are under the same frame (IGb08) nominally.

The combined daily terrestrial frame solutions between 1994.0 and 2015.2 are used to generate cumulative solutions with different strategies in this study. Unlike solutions from any individual analysis center, the combination of multi-analysis centers is more reliable and robust since the noise can be averaged out partly, moreover, having many more sites included in the combination is another advantage for the combined daily solution. Nine different analysis centers (ACs) participated in the daily terrestrial frame solution combination or comparison in the second reanalysis. The combined daily terrestrial frame solution includes station coordinates, Earth rotation parameter (ERP) and apparent geo-center motion parameters, as well as the full covariance matrix and the corresponding a priori information. The daily combined solution is aligned to the IGb08 reference frame using the core stations with no-net-rotation (NNR) and no-net-translation (NNT) constraints. All the information is stored in the form of solution independent exchange (SINEX) files. The inter-AC agreement performance of station coordinates is 1.5 mm and 4 mm for the horizontal and vertical components, respectively. Regarding the pole coordinates, pole rates and calibrated length of day (LOD) parameter, the agreement between ACs is 25–40 *μ*as, 140–200 *μ*as/d and 8–20 *μ*s/d, respectively. A detailed description of the combination method for the second reprocessed SINEX daily solutions can be found in Rebischung’s study [28].

### 2.2. GNSS Station Position Series Processing Strategy

The daily combination derived from the Equation (11) in Rebischung’s study [28] includes station coordinates, ERP and geo-center motion parameters. These different solutions in daily SINEX files are stacked simultaneously to obtain a cumulative solution and the corresponding formulae are used as:

(1) $$\begin{array}{c} \;x_{i}^{s}\left(t_{k}\right)+v_{i}^{s}\left(t_{k}\right)=x_{i}^{c}\left(t_{0}\right)+{\dot{x}}_{i}^{c}\left(t_{k}-t_{0}\right)+T_{t_{k}}+R_{t_{k}}x_{i}^{c}\left(t_{0}\right)+\\ \;\overset{m}{\underset{p=1}{\sum }}o_{p}H\left(t_{k}-t_{p}^{o}\right)\;+\overset{n}{\underset{j=1}{\sum }}a_{j}sin\left\{w_{j}\left(t_{k}-t_{0}\right)+\varphi_{j}\right\} \end{array}$$

And

(2) $$\begin{array}{c} \;x_{GC}^{s}\left(t_{k}\right)+v_{GC}^{s}\left(t_{k}\right)=x_{GC}^{c}\left(t_{k}\right)+T_{t_{k}}\\ \;x_{p}^{s}\left(t_{k}\right)+v_{xp}^{s}\left(t_{k}\right)=x_{p}^{s}\left(t_{k}\right)+R_{t_{k}}^{Y}\\ \;y_{p}^{s}\left(t_{k}\right)+v_{yp}^{s}\left(t_{k}\right)=y_{p}^{c}\left(t_{k}\right)+R_{t_{k}}^{X}\\ \;{\dot{x}}_{p}^{s}\left(t_{k}\right)+v_{xpr}^{s}\left(t_{k}\right)={\dot{x}}_{p}^{s}\left(t_{k}\right)\\ \;{\dot{y}}_{p}^{s}\left(t_{k}\right)+v_{ypr}^{s}\left(t_{k}\right)={\dot{y}}_{p}^{s}\left(t_{k}\right)\\ \;LOD^{s}\left(t_{k}\right)+v_{LOD}^{s}\left(t_{k}\right)=LOD^{c}\left(t_{k}\right) \end{array}$$

where

- $x_{i}^{l}\left(t_{k}\right)$, $x_{GC}^{l}\left(t_{k}\right)$, $x_{p}^{l}\left(t_{k}\right)$, $y\left(t_{k}\right)$, ${\dot{x}}_{p}^{l}\left(t_{k}\right)$, ${\dot{y}}_{p}^{l}\left(t_{k}\right)$ and $LOD^{l}\left(t_{k}\right)$ are coordinate vectors of station i, geocenter motion in Cartesian coordinates system and ERPs of daily (l=s) and stacking (l=c) solutions, respectively. $x_{i}^{c}\left(t_{0}\right)$ and ${\dot{x}}_{i}^{c}$ are initial coordinates and the velocity of station i at the reference epoch $t_{0}$. $v_{i}^{s}\left(t_{k}\right)$, $v_{GC}^{s}\left(t_{k}\right)$, $v_{xp}^{s}\left(t_{k}\right)$, $v_{yp}^{s}\left(t_{k}\right)$, $v_{xpr}^{s}\left(t_{k}\right)$, $v_{ypr}^{s}\left(t_{k}\right)$ and $v_{LOD}^{s}\left(t_{k}\right)$ denote the residuals for the observations of daily coordinates, geo-center motion parameters and ERPs.
- $T_{t_{k}}$ and $R_{t_{k}}$ denote the translation and rotation parameters of similarity transformation between daily and stacking coordinates, which can be expressed as matrices
  (3) $$T_{t_{k}}=\left[\begin{array}{c} T_{t_{k}}^{X}\\ T_{t_{k}}^{Y}\\ T_{t_{k}}^{Z} \end{array}\right],\;R_{t_{k}}=\left[\begin{array}{ccc} 0 & -R_{t_{k}}^{Z} & R_{t_{k}}^{Y}\\ R_{t_{k}}^{Z} & 0 & -R_{t_{k}}^{X}\\ -R_{t_{k}}^{Y} & R_{t_{k}}^{X} & 0 \end{array}\right]$$
- $o_{p}$ is the offset of the coordinate series at epoch $t_{p}^{o}$, $H\left(t_{k}-t_{p}^{o}\right)$ is the Heaviside step function and can be expressed as
  (4) $$\;H\left(t_{k}-t_{p}^{o}\right)=\{\begin{array}{c} 1,\;\;\;\;\;t_{k}\geq t_{p}^{o}\;\\ 0,\;\;\;\;\;t_{k}<t_{p}^{o} \end{array}$$
- $a_{j}$, $w_{j}$ and $\varphi_{j}$ are the amplitude, angular rate and phase of the optional periodic signal j with frequency $f_{j}$ and $w_{j}=2\pi f_{j}$.

The scale parameter is not applied in the stacking model since inclusion of a scale factor can permit the aliasing of surface loading deformation [29,30]. But a solution with the scale parameter considered additionally is obtained for comparison in this paper.

Intermittent offsets happening in the coordinate series would degrade the uncertainty of the position and velocity estimation at the reference epoch. Moreover, a misleading understanding of noise in the coordinate series would be caused by the offsets [31]. The work of offset detection plays an important role in estimating station velocities. Compared to the automated methods, Gazeaux et al. [32] showed that the manual methods of offset detection had a more promising performance in their experiments and indicated that it was difficult to interpret the sub-mm/yr velocities accurately due to the existing offsets. Offsets in the position series can be induced by many factors, including seismic events, equipment, environment or metadata changes and monument disruptions and have different patterns. Unlike offsets caused by many factors, the post-seismic deformation is caused by major earthquakes and it is always nonlinear. Altamimi et al. [33] applied a post-seismic deformation model to the position time series of stations near earthquake colocations. The same model was used in this study before daily solution stacking. Moreover, discontinuity information for the position and velocity was also adopted directly from IGS and the authors assume the residual post-seismic deformation and the undetected offsets have the same effects on different solutions.

Six solutions with different strategies, listed in Table 1, were obtained in this paper. The reasons for selecting these solutions were as follows. First, traditional methods to obtain the seasonal signals from the position series were implemented sequentially by station; the correlations within different stations were neglected; this is the solution unit_xxx_s2, with the unit weight matrix and no transformation parameters considered. The solutions with a full weight matrix taken from the covariance matrix in SINEX files were computed and compared to the solution with a unit weight matrix (unit_tr_s2 versus cova_tr_s2). Second, to check the impact of Helmert parameters on the seasonal signals, the cumulative solution with these parameters (three translation, three rotation and one scale parameter) is obtained and compared. Third, to evaluate the contribution of environmental loading models to the GNSS position time series, solutions with and without periodic signals, were stacked and compared (cova_tr_s0 versus cova_tr_s2). Although observations with at least 25.4-year span is required to separate the annual (1 cpy) and draconitic (1.04 cpy) periodic signals, the solution with draconitic signals up to second harmonics, in addition to seasonal signals, is obtained for comparison, which is labeled as cova_tr_s2d2 in this paper. Regarding all solutions with transformation parameters estimated in the stacking model, a loose constraint (0.1 m) is adopted and no other constraints are applied.

**Figure 1:**
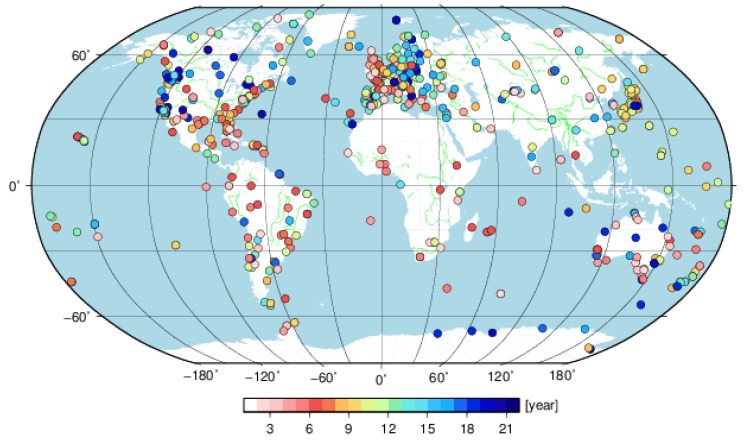
The global distributed stations with different observation time spans.

To resolve frequencies higher than 1.0 cpy, stations with observation time spans less than two years (or 730 observations) are eliminated in the normal equation for each SINEX solution. An iterative outlier detection and deletion process is applied in the data preprocess until no observations have residuals larger than 5-sigma for each station. Moreover, stations with a data gap larger than 20% are also excluded. As a result, 647 stations in total remain in the final stacking solution. Figure 1 shows the distribution of the GNSS sites with different observation time spans. There are 33 of 647 stations with observation time spans less than three years. The median observation time span is 9.9 years and the number of stations with observation time spans larger than 12 years is 192. There are also 14 stations with observation time spans more than 20 years.

## 3. Environmental Loading Deformation

As seasonal signals can be predicted by an environmental loading model, displacements of sites in the center of the Earth’s figure (CF) frame are collected from the École et Observatoire des Sciences de la Terre (EOST) loading service. Three contributed parts are included in the displacements: (1) Regarding the displacements caused by atmospheric loading, there are three kinds of products provided by EOST Loading Service. Two kinds of products with a time resolution of three hours are estimated from surface pressure provided by the European Center for Medium Range Weather Forecasts (ECMWF) but with different models for the ocean response. One assumes an inverted barometer and the other uses a TUGO-m barotropic model [34]. The displacements caused by atmospheric loading also can be estimated from ECMWF reanalysis, assuming an inverted barometer ocean response to pressure forcing. The atmospheric loading products assuming the inverted barometer using the ECMWF operational model is used in this study; (2) The displacements induced by non-tidal oceanic loading are derived from the ocean bottom pressure gained by the Estimating Circulation and Climate of the Ocean (ECCO) [35,36] with daily and 0.25 degree resolution; (3) The displacements caused by hydrology loading (soil moisture, snow and canopy water) are derived from the Global Land Data Assimilation System (GLDAS) with three hour and 0.25 degree time and spatial resolution [37]. Meanwhile, the permanent ice covered regions (Greenland, Alaska, mountain glaciers and so on) have been masked out. The AOH in this paper denotes the sum of the above three loading deformations.

First, the displacements of atmospheric pressure loading and hydrology loading are averaged into daily solutions, separately. Then the combined impacts of different loading contributors are summed. There is a secular trend in the summed loading displacement series [9,13], which relates to the method of the loading model establishment or real geophysical signal. As the seasonal signals are focused in this paper, the authors detrended the series of sum displacement. Finally, annual plus semi-annual signals (sine and cosine terms) were extracted by fitting the detrended series. Even though the displacement series of atmospheric loading covering the time before 2 November 2000 are not available, it is sufficient to compute the average amplitude and phases of annual and semi-annual signals using the data with a time span from 2001 to 2015.

## 4. Assessment of the Seasonal Signals within Different Strategies

The seasonal signals derived from the GNSS stacking solutions with different strategies and environmental loading model are compared. The effects of the Helmert transformation parameters and weight matrices on seasonal signals are analyzed.

### 4.1. Effect of Helmert Transformation Parameters on Seasonal Signals

**Figure 2:**
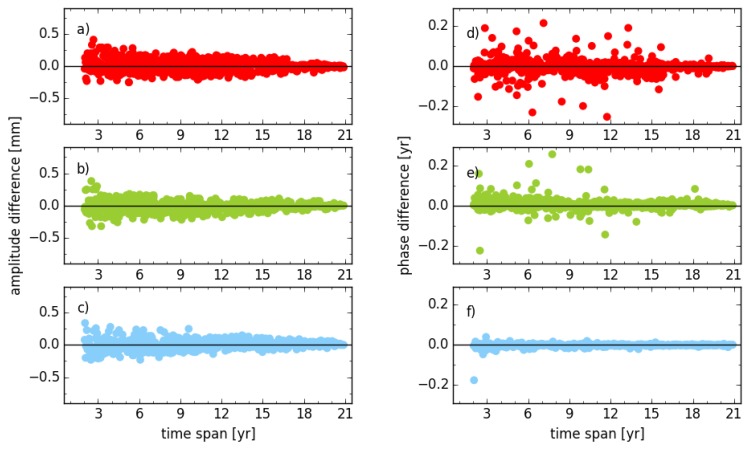
Amplitude and phase differences of the annual signal between solution unit_xxx_s2 and unit_tr_s2 for stations with different observation time spans; (**a**,**d**) for east; (**b**,**e**) for north; (**c**,**f**) for vertical components.

Figure 2 illustrates the impact of transformation parameters (three translation and three rotation parameters) on the amplitude and phase of the annual signal. The phases displayed in the figure are divided by the angular frequency (2π). Considering amplitude and phase, the difference between with and without six transformation parameters estimated shows a convergence to zero with the increasing observation time span. The RMS of amplitude changes from 0.14 mm, 0.18 mm and 0.14 mm to 0.02 mm, 0.03 mm and 0.03 mm for the east, north and vertical components, respectively, for stations with observation time spans of less than three years (33 stations) to more than 19 years (36 stations). Meanwhile, the RMS of the annual phase for the same components changes from 18°, 20° and 12° to 3°, 2° and 1°, respectively. Concerning the whole time span, the RMS of the amplitude is 0.10 mm, 0.09 mm and 0.07 mm, while the RMS for the phase is 17°, 11° and 3° for east, north and vertical components, respectively.

**Figure 3:**
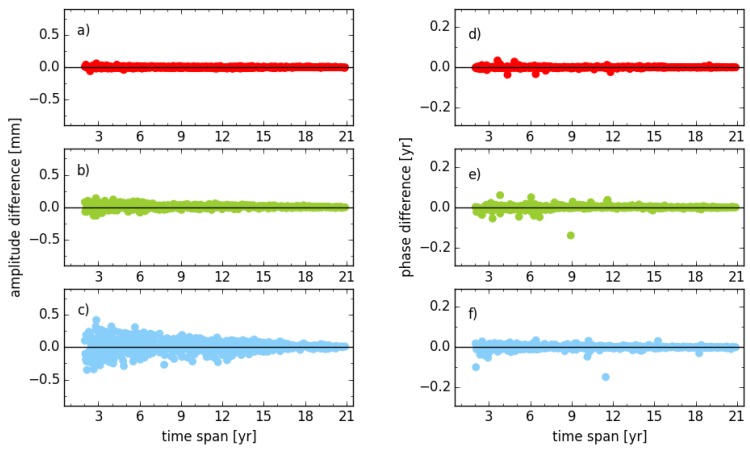
Amplitude and phase differences of annual signal between solution unit_tr_s2 and unit_trs_s2 for stations with different observation time spans; (**a**,**d**) for east; (**b**,**e**) for north; (**c**,**f**) for vertical components.

Compared to the solution neglecting the scale parameter (solution unit_tr_s2), the amplitude of the annual signal with an additional scale parameter considered (solution unit_trs_s2) almost has no change for the horizontal components (Figure 3). The RMS of all stations is 0.01 mm and 0.03 mm for the east and north components, respectively. However, the RMS for the vertical is about three times (0.1 mm) larger than the one for the north component. A Similar result is obtained by comparison between solution cova_tr_s2 and cova_trs_s2 but not shown here. It indicates that the scale parameter would absorb part of the common seasonal signals, though the seasonal signals have already been parameterized in the stacking model. Therefore, the scale parameter neither should be estimated in the stacking model nor considered in the daily combination [28]. Regarding the phase of the annual signal, the RMS of all stations is about 1°, 4° and 4° for the east, north and vertical components, respectively. Conversely, the scale parameter has negligible impact on the amplitude difference of the vertical component when the time span of the position time series increasing, the RMS reduces from 0.21 mm to 0.02 mm. However, an RMS of 0.06 mm is observed still for the series with a time span larger than 11 years, which motivates the authors to obtain stacking solutions without considering the scale parameter for further comparison.

### 4.2. Effect of Different Weight Strategies on Seasonal Signals

**Figure 4:**
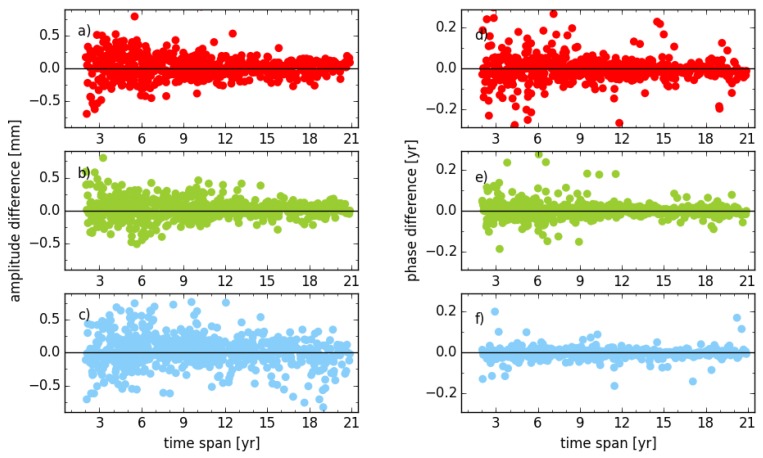
Amplitude and phase differences of annual signal between solution unit_tr_s2 and cova_tr_s2 for stations with different observation time spans; (**a**,**d**) for east; (**b**,**e**) for north; (**c**,**f**) for vertical components.

To evaluate the impact of a weight matrix on seasonal signals, the differences of amplitude and phase between solutions, with unit and full weight matrix (solution unit_tr_s2 versus cova_tr_s2), are illustrated in Figure 4. Even with an observation time span of more than 11 years, the maximum amplitude difference still can reach 0.53 mm, 0.41 mm and 1.14 mm for the east, north and vertical components, respectively, while the maximum phase difference is 113°, 64° and 62° for the three components. The RMS of amplitude difference for all stations caused by a different weight matrix is 0.18 mm, 0.15 mm and 0.27 mm for the east, north and vertical components, respectively and the corresponding value for phase is 26°, 7° and 10°. These indicate the weight matrix plays a more important role than the Helmert parameters for the global network and should be considered carefully. It also can be found that there are fourteen stations with amplitude difference larger than 0.5 mm for the vertical component, even though the observation time span is more than 15.0 years. The average amplitude of the annual signal for those stations is 4.5 mm (solution cova_tr_s2), which is about the 80th percentile amplitude of all stations. The large amplitude of seasonal signals and weight matrices might be the possible reason for these stations with large amplitude biases thus, further investigation is needed.

**Figure 5:**
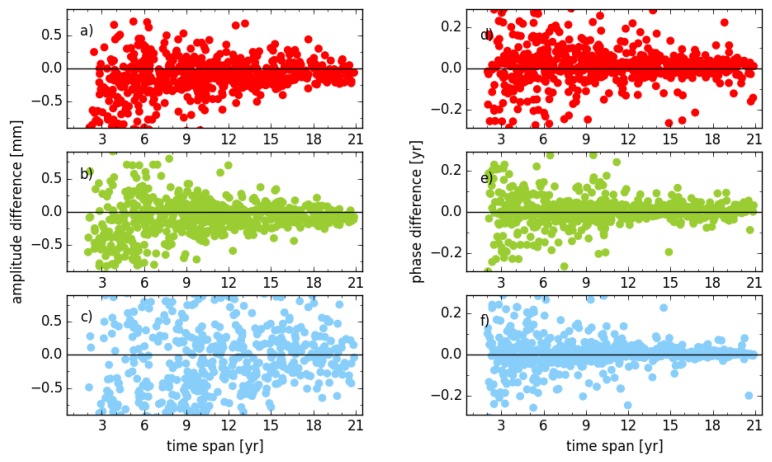
Amplitude and phase differences of annual signal between solution cova_tr_s2 and cova_tr_s2d2 for stations with different observation time spans; (**a**,**d**) for east; (**b**,**e**) for north; (**c**,**f**) for vertical components.

Compared to the cumulative results with unit weight matrices used in the daily solutions stacking, the estimated parameters are more rigorous with the covariance information of observation considered. As a result, the authors take the solutions with the full weight matrix for further comparison. Figure 5 shows the difference with and without the up to the second draconic signals parameterized in addition to the seasonal signals. An obvious difference is found for the estimated annual amplitude and phase special for the stations with a time span less than 15 years. However, both the RMS of annual amplitude and phase become a little more stable when the observation time span is larger than 15 years. The RMS of amplitude for all time spans is 0.8 mm, 0.6 mm and 1.7 mm for east, north and vertical component, respectively and decrease to 0.2 mm, 0.1 mm and 0.4 mm when the time span increases to 15 years. The RMS of phases also changes from 43°, 33° and 31° to 25°, 16° and 10° for the three components. This might indicate that the annual signal and GPS draconic signal can be separated marginally with a time span of more than 15 years.

### 4.3. Comparison of the Estimated Seasonal Variations with Environmental Loading

To assess the level of agreement between seasonal signals derived from GNSS and AOH, the RMS misfit of seasonal signals between GNSS and AOH is computed using the formula:

(5)  RMSmisfit={1n∑i=1∞|di|2}12 di=Aijgnss{isin(φijgnss)+cos(φijgnss)}−Aijaoh{isin(φijaoh)+cos(φijaoh)}

where *n* is the number of selected stations, $A_{ij}^{gnss}$ and $\varphi_{ij}^{gnss}$ are the annual amplitude and phase derived from station position time series, respectively. $A_{ij}^{aoh}$ and $\varphi_{ij}^{aoh}$ are the annual amplitude and phase derived from the AOH model, respectively.

Table 2 lists the RMS misfit of seasonal signals between the GNSS and AOH models. The solution cova_tr_s2d2 with the additional GPS draconic signals and up-to-the-second harmonics estimated, shows the worst performance. Therefore, it could be caused by the correlation between seasonal and draconic signals, which is also validated in Figure 5. According to the Rayleigh criterion, the series with an at least 25.4-year time span is required to separate periodic signals of 1.0 cpy and 1.04 cpy. There are even less than 200 stations with an observation time span larger than 12 years to resolve the signals of 2.0 cpy and 2.08 cpy. Thus, the seasonal and GPS draconic signals of low frequencies are easily aliased to each other when both are considered in a stacking model using a position time series with a finite time span. The solution cova_tr_s2 seems to perform the best for the seasonal signals, except for the east component of the annual signal. Refarding the four solutions with the same periodic signals parameterized in the stacking model (solution unit_xxx_s2, unit_tr_s2, unit_trs_s2 and cova_tr_s2), the difference is less than 0.03 mm for annual signal and it is smaller than 0.1 mm for the semi-annual signal. The solution with full weight and six Helmert transformation parameters (no scale parameter) are used for further comparison (solution cova_tr_s2).

**Figure 6:**
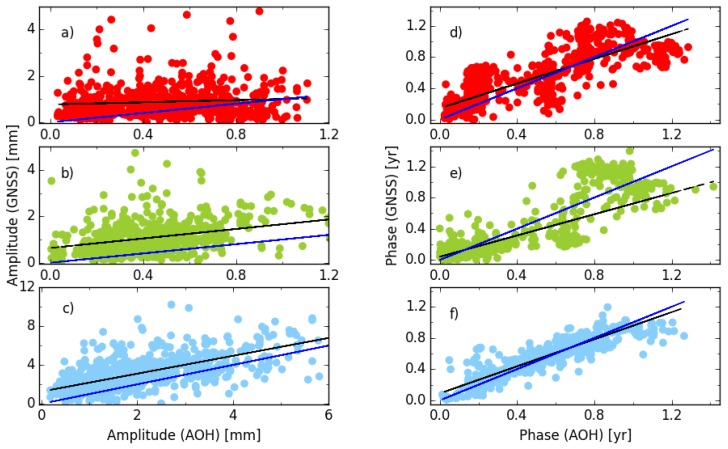
The agreement performance of annual signal between GNSS and AOH; (**a**,**d**) for east; (**b**,**e**) for north; (**c**,**f**) for vertical components. The phase is divided by 2π and, to keep the absolute phase difference less than 0.5 year, the values out of the range from −0.5 to 0.5 year are added one cycle and not used for fitting. The black line is the linear fitting result between GNSS and AOH and the blue line shows 1:1 perfect agreement.

Figure 6 compares the amplitude and phase of the annual signal between solution cova_tr_s2 and the AOH model using 647 global stations. The black line is the linear fitting between the GNSS and AOH and the blue line shows 1:1 perfect agreement. The phases displayed in the figure are divided by the angular frequency (2π) and the phase differences are kept at less than one half period (0.5 year). The annual amplitude of the vertical component is larger than the one of the horizontal component for most stations. The median amplitudes derived from the AOH model are 0.49 mm, 0.44 mm and 1.94 mm for the east, north and vertical components, respectively. Meanwhile, most stations have larger annual amplitudes derived from the GNSS position series than those obtained from the AOH model. There are 72.5%, 89.3% and 81.2% of stations with larger GNSS-derived annual amplitudes in 647 stations for the east, north and vertical components, respectively. The median annual amplitudes obtained from the cova_tr_s2 solution are 0.72 mm, 0.93 mm and 2.95 mm, respectively, for the east, north and vertical component and the median of absolute amplitude differences between the GNSS and AOH are 0.40 mm, 0.54 mm and 1.06 mm for the three components. The median ratio factors of annual amplitude between the cova_tr_s2 solution and AOH are 1.7 mm, 2.3 mm and 1.5 mm for the east, north and vertical component, respectively.

**Figure 7:**
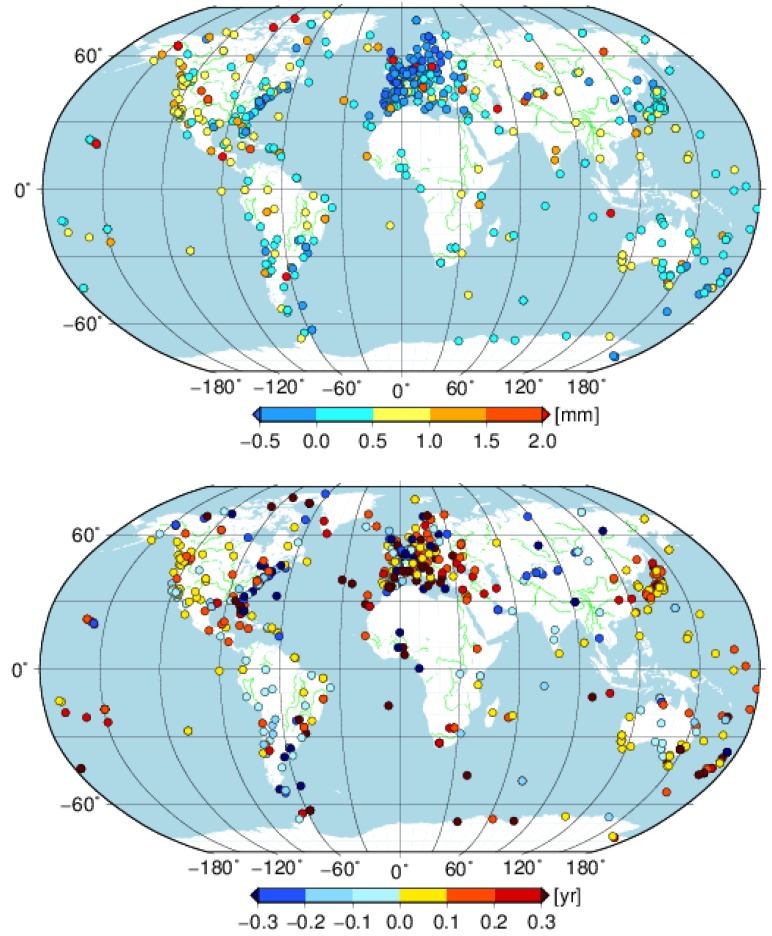
The amplitude (**top panel**) and phase (**bottom panel**) differences of annual signal between GNSS and AOH for east component for the selected 647 global stations.

Moreover, it is interesting that stations with smaller annual amplitudes for the east component derived from GNSS are mainly distributed over the area with a longitude range from −30° to 40° and a latitude from 30° to 90° (Figure 7). There are 128 of 192 stations (67%) in this area, which account for 70% of all the stations with larger AOH annual amplitudes. However, there are only 38 and 61 of 194 (20% and 31%) stations with larger AOH annual amplitudes in this area for the north and vertical components (not shown in the figure), respectively. The different annual amplitude agreement performances between the GNSS and AOH for the three components are also found from the fitting lines. The fitting lines have slopes of 0.23 ± 0.12, 1.02 ± 0.13 and 0.92 ± 0.09 for the east, north and vertical components, respectively. It indicates that the annual amplitude difference in the east has more regional distribution characteristics. A worse agreement result for east component was also shown by Collilieux [38] using the first reprocess IGS weekly combinations. Excluding the stations in the area described above, the slope of the east component becomes 0.87 ± 0.15. A further investigation is needed to scrutinize the different performances of the east annual amplitude over this area.

Regarding the phase of the annual signal, the best agreement performance between the GNSS and AOH models, is found for the vertical component. The median absolute difference is 0.05 year (18°) and the slope of fitting line is 0.87 ± 0.08. However, the differences between the GNSS and AOH are more scattered for the remaing two components. Although the slopes are 0.79 ± 0.08 and 0.68 ± 0.12, the median of the absolute phase differences is 0.14 and 0.11 year (50° and 40°) for the east and north components, respectively. Therefore, since the seasonal displacement caused by mass loading is more obvious for the vertical than the horizontal components, the small horizontal annual signals in the position series are more easily contaminated by unmodeled or/and residual modeled errors related to the GNSS technique.

**Figure 8:**
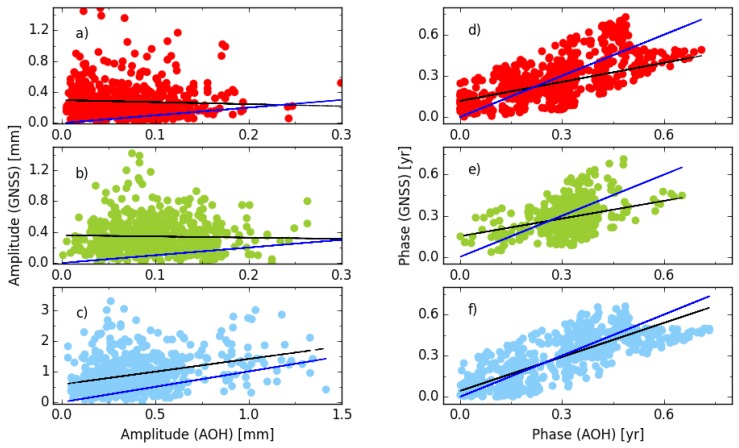
The agreement performance of semi-annual signal between GNSS and AOH, (**a**,**d**) for east; (**b**,**e**) for north; (**c**,**f**) for vertical component. The phase is divided by 4π and to keep the absolute phase difference less than 0.25 year, the values out of the range from −0.25 to 0.25 year are added by 0.5 year and not used for fitting.

Figure 8 compares the amplitude and phase of the semi-annual signal between the cova_tr_s2 solution and the AOH model using 647 global stations. The black line is the linear fitting and the blue line shows the 1:1 agreement between the GNSS and AOH. The phase is divided by 4π to keep the absolute phase difference less than 0.25 year and the values out of the range from −0.25 to 0.25 year are added by 0.5 year. The semi-annual signal has smaller amplitudes than the ones of the annual signal, the median amplitudes derived GNSS are 0.22 mm, 0.29 mm and 0.76 mm, while the amplitudes predicted by the AOH model are 0.07 mm, 0.09 mm and 0.32 mm for the east, north and vertical components, respectively. Moreover, the phases of the north component are centered at 0.35 year (252°). The median absolute amplitude difference of the semi-annual signals between the GNSS and AOH are 0.15, 0.20 and 0.42 mm for east, north and vertical component, respectively. For the phases of semi-annual signals, the median differences between GNSS and AOH are 0.10, 0.06 and 0.09 year (i.e., 72°, 43° and 65°) for the east, north and vertical components, respectively.

## 5. Impact of Seasonal Signals on Position and Velocity

To assess the contribution of parameterization in the stacking model or the AOH model to the GNSS position time series, the WRMS reduction of position residuals is calculated with the same formulas as Jiang’s study [13]:

$$\;{\mathrm{WRMS}}_{\mathrm{gnss}}\left(\%\right)=100\times \frac{{\mathrm{WRMS}}_{{\mathrm{cova}}_{{\mathrm{tr}}_{s0}}}-{\mathrm{WRMS}}_{{\mathrm{cova}}_{{\mathrm{tr}}_{s2}}}}{{\mathrm{WRMS}}_{{\mathrm{cova}}_{{\mathrm{tr}}_{s0}}}}$$

(6) $$\;{\mathrm{WRMS}}_{\mathrm{aoh}}\left(\%\right)=100\times \frac{{\mathrm{WRMS}}_{\mathrm{cova}\_\mathrm{tr}\_s0}-{\mathrm{WRMS}}_{\mathrm{cova}\_\mathrm{tr}\_s0-\mathrm{aoh}}}{{\mathrm{WRMS}}_{\mathrm{cova}\_\mathrm{tr}\_s0}}$$

where ${\mathrm{WRMS}}_{\mathrm{cova}\_\mathrm{tr}\_s0}$ and ${\mathrm{WRMS}}_{\mathrm{cova}\_\mathrm{tr}\_s2}$ are the WRMS of the GNSS stacking solution, with and without considering the seasonal signals parameterized in the stacking model, respectively. ${\mathrm{WRMS}}_{\mathrm{cova}\_\mathrm{tr}\_s0-\mathrm{aoh}}$ is the WRMS of solution cova_tr_s0 residuals with seasonal displacements corrected by the AOH model.

Regarding solution cova_tr_s2, all the stations have their WRMS reduced compared to cova_tr_s0, except the seven and three stations have no WRMS change in the east and north direction. Compared to the solution cova_tr_s0, when the seasonal signal parameters are estimated in the stacking solution (cova_tr_s2), the mean WRMS is reduced from 1.95 mm, 1.95 mm and 6.07 mm to 1.78 mm, 1.71 mm and 5.41 mm, with the percentage of WRMS reduction approximately 11%, 15% and 14% for the east, north and vertical components, respectively.

**Figure 9:**
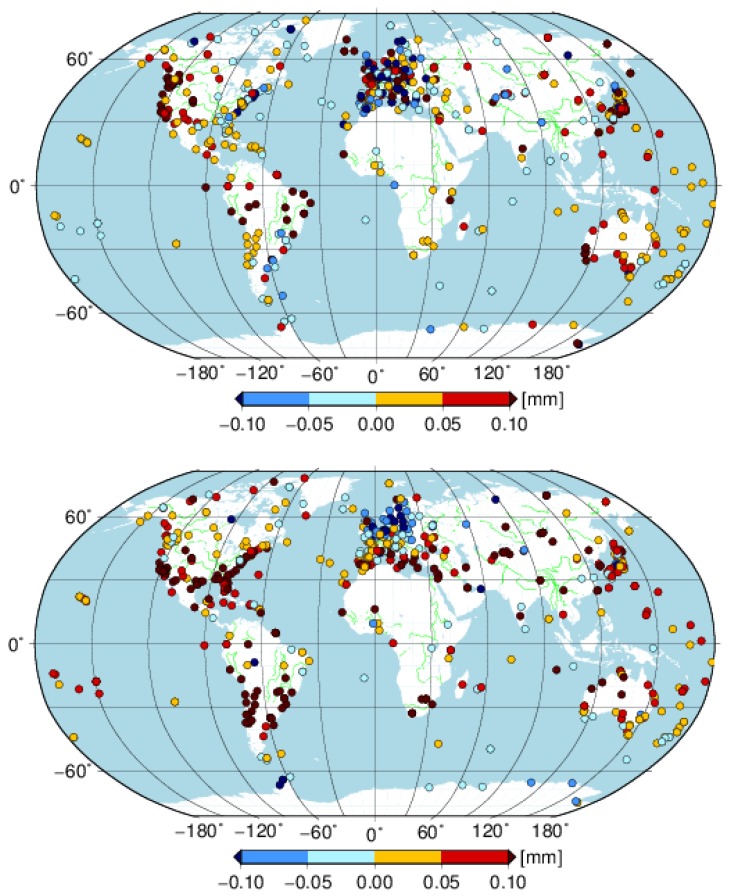

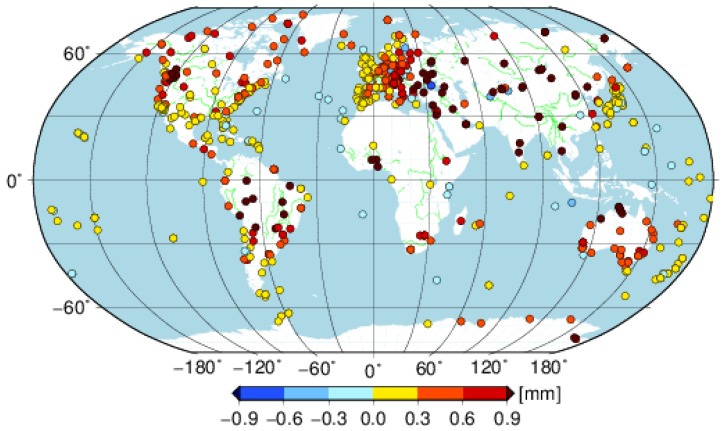
WRMS difference between cova_tr_s0 without and with seasonal displacement corrected by AOH model for the selected global stations for the east (**top panel**); north (**middle panel**) and vertical (**bottom panel**) component.

Figure 9 illustrates the WRMS difference between cova_tr_s0 and cova_tr_s0 with seasonal displacements corrected by AOH for each station. There are 62%, 72% and 90% of 647 stations with WRMS reduced when the residuals of solution cova_tr_s0 are corrected by the AOH model. A better performance of the vertical component than the results of Jiang, W. et al. [13] is found, whereas, in that study, three different environmental loading models were compared and the best performance was 74% of 233 stations with the WRMS reduced in the vertical direction. The better performance in the current study might be attributed to the latest data reanalysis methodology and the model in the second IGS reprocessing campaign (http://acc.igs.org/reprocess2.html). While the residuals of cova_tr_s0 are corrected by the AOH, the corresponding mean WRMS reduction of all stations is 0.03 mm, 0.07 mm and 0.41 mm for the east, north and vertical components, respectively. Taking ${\mathrm{WRMS}}_{\mathrm{aoh}}/{\mathrm{WRMS}}_{\mathrm{gnss}}$ as the contribution of the AOH model to the GNSS WRMS reduction, then the seasonal signals predicted by the AOH model could explain 18%, 29% and 62% of the seasonal signals derived from the GNSS position series for the east, north and vertical components, respectively.

**Figure 10:**
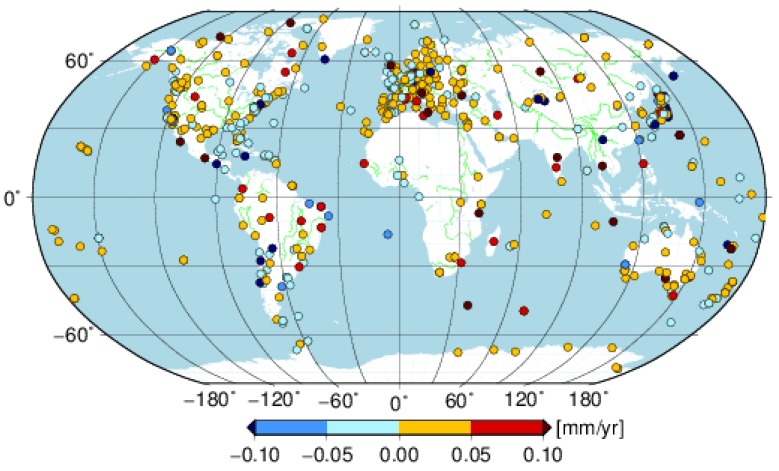

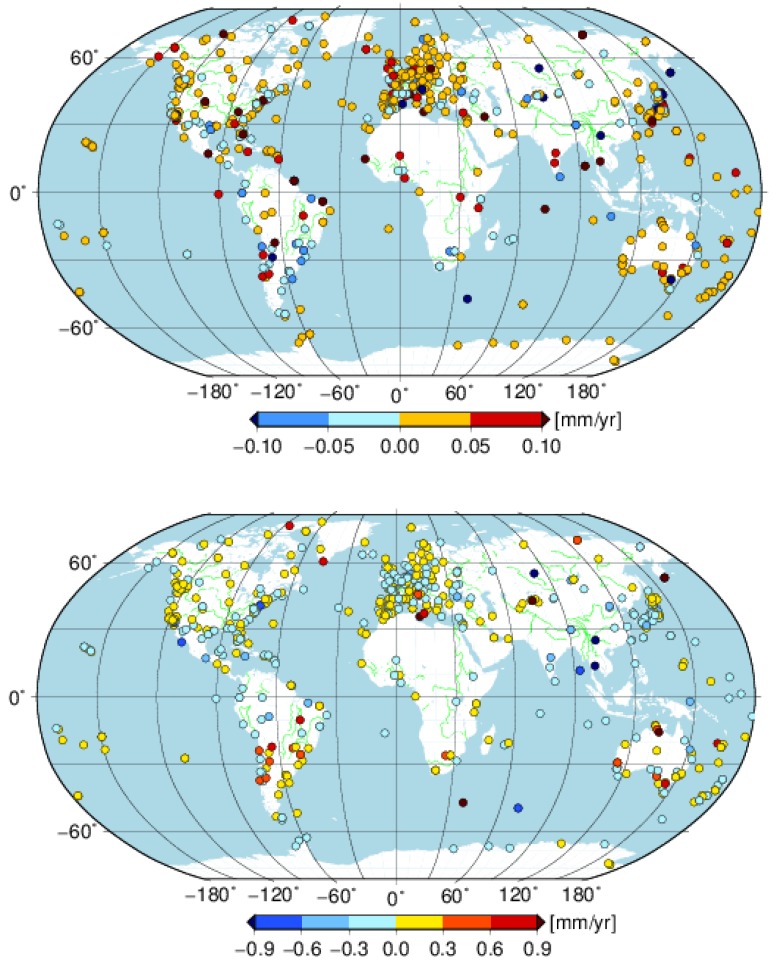
Velocity difference between solution cova_tr_s0 and cova_tr_s2 for east (**top panel**); north (**middle panel**) and vertical (**bottom panel**) component.

To compare the velocity parameters between with and without seasonal signals parameterized in the stacking model, Figure 10 illustrates the velocity difference between solution cova_tr_s0 and cova_tr_s2 of the selected 647 global distributed stations with different observation time spans. Concerning the horizontal velocity difference, most stations have a velocity bias of less than 0.1 mm/yr. There are 82% and 78% of stations with a velocity bias falling into the range −0.05 to 0.05 mm/yr for the east and north components, respectively and 91% of stations with an absolute velocity bias less than 0.1 mm/yr for the horizontal component. Generally, the shorter the observation span (see Figure 1), the greater the velocity bias obtained for the selected stations in this paper. The large amplitude of periodic signals could also enlarge the bias [39], which is the case for the velocity bias in the vertical direction. Regarding vertical component, the percentage of stations with velocity bias falling into the range −0.05 to 0.05 mm/yr is only 54% and 90% of stations have an absolute bias less than 0.3 mm/yr.

**Figure 11:**
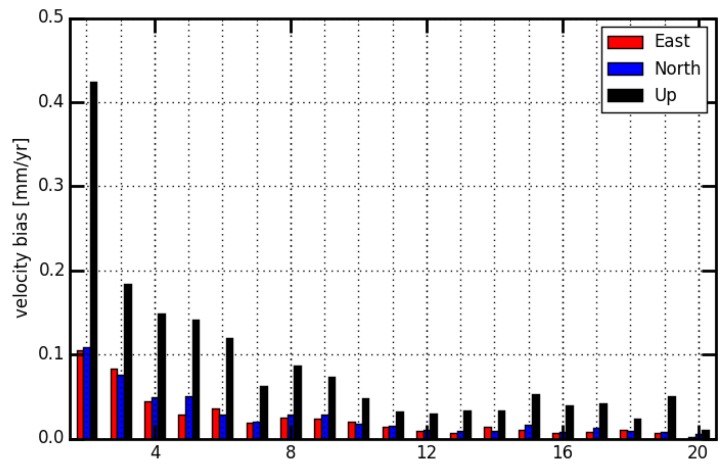
Mean of absolute velocity bias between solution cova_tr_s0 and cova_tr_s2.

Figure 11 illustrates the average absolute velocity difference between without and with seasonal signals considered in the deterministic model (cova_tr_s0 versus cova_tr_s2). The velocity estimates could be affected by seasonal signals. Since larger amplitude seasonal signals were observed in the GNSS position series of the vertical component than the ones of the horizontal component, the vertical velocity difference is about 2–4 times larger than those in the horizontal. The horizontal velocity difference is less than 0.05 mm/yr, when the observation time span is more than 5 years. However, the velocity difference for vertical component between solution cova_tr_s0 and cova_tr_s2 is still larger than 0.1 mm/yr until the observation time span is greater than 7 years.

## 6. Discussion and Conclusions

Seasonal signals are derived from both the IGS second reprocessing daily terrestrial frame solution and the environmental loading model in this contribution. Different weight strategies and Helmert transformation parameters are adopted in the time series stacking model. Now the authors can answer the questions proposed in Section 1. Does the weight matrix or the transformation parameters have an impact on the seasonal signals? Compared to seasonal signals estimated from each coordinate time series separately, the Helmert transformation parameters (translation and rotation) can induce a root mean square of 0.1 mm for both the horizontal and vertical components. The scale parameter causes an additional 0.1 mm for the vertical component and should be considered carefully in the stacking model. Moreover, the difference closes to zero with an increased observation time span. To compare unit and full weight matrices in the stacking model for all stations, the different weight matrices can lead to a scatter of annual signals of 0.18 mm, 0.15 mm and 0.27 mm for the east, north and vertical components, respectively and to the scatter of the annual phase of 26°, 7° and 10° for the three components. So the weight matrices for observations should be carefully considered in the global network. Meanwhile, the decreasing scatter of both annual amplitude and phase is found with an increased observation time span.

Should researchers estimate the draconic signals simultaneously when extracting the seasonal signals using the position time series with a finite time span? Compared to the cumulative solution with only seasonal signals estimated, the additional draconic signals up-to-second harmonics can cause a scatter of 0.8 mm, 0.6 mm and 1.7 mm for the east, north and vertical components, respectively. Increasing the observation time span to 15 years, the scatter reduces to 0.2 mm, 0.1 mm and 0.4 mm for the east, north and vertical components, respectively. There are only about 200 stations with observation time span larger than 12 years to separate periodic signals of 2.0 cpy and 2.08 cpy. Moreover, it is not reliable to resolve the signals of 1.0 cpy and 1.04 cpy using the IGS reprocessed solutions with maximum time spans of 22 years. The seasonal and GPS draconic signals of low frequencies might leakage into each other when both signals are considered in stacking model using position time series with a finite time span. This is also validated by the worse agreement with environmental loading displacements for seasonal signals than the results from solutions without draconic signals estimated in the deterministic model.

How much can the environmental loading model contribute to the seasonal signals derived from the GNSS position series? Seasonal signals derived from the GNSS and environmental loading model are shown in this study. The median amplitude of annual and semi-annual signals derived from GNSS is about 1.5–3.2 times larger than the results of the loading model. Both the GNSS and environmental loading model found the annual amplitude is approximately 2.1–2.9 times larger than the semi-annual amplitude. Compared to the cumulative solution without any periodic parameters estimated, when the annual and semi-annual signals are considered in the position series stacking, the WRMS reduction is about 11%, 15% and 14% for the east, north and vertical components, respectively. When the residuals of the solution with no seasonal signals considered in the stacking model are corrected by the environmental loading model, the percentage of stations with the WRMS reduced is 62%, 72% and 90% for east, north and vertical components, respectively. Taken from the ratio of ${\mathrm{WRMS}}_{\mathrm{aoh}}$ to ${\mathrm{WRMS}}_{\mathrm{gnss}}$, it indicates that the environmental loading model can explain 18%, 29% and 62% of seasonal signals in the GNSS coordinate time series for the east, north and vertical components, respectively.

Compared to the velocity estimated with seasonal signals considered in the deterministic model, the authors found that an observation time span greater than seven years should be needed for a linear model to obtain a velocity estimate with a bias less than 0.1 mm/year. The authors also should be clear that all cumulative solutions obtained from the presented stacking model are under the assumption of white noise for the position time series. Seasonal signals and the stochastic model, as well as the velocity estimates would have a mutual impact on each other [40] and the color noise can be found in the GNSS position time series [28]. A comprehensive analysis of parameters in the deterministic model and stochastic model is needed in future but is beyond the scope of this study.

## Figures and Tables

**Table 1:** Solutions with different strategies for comparison.

Solution	Weight Matrix	Transformation Parameter	Periodic Signals
unit_xxx_s2	Unit	No	1 cpy, 2 cpy
unit_tr_s2	Unit	Translation, rotation	1 cpy, 2 cpy
unit_trs_s2	Unit	Translation, rotation, scale	1 cpy, 2 cpy
cova_tr_s0	Full	Translation, rotation	None
cova_tr_s2	Full	Translation, rotation	1 cpy, 2 cpy
cova_tr_s2d2	Full	Translation, rotation	1 cpy, 2 cpy, 1.04 cpy, 2.08 cpy

**Table 2:** RMS misfit of GNSS position series stacking solutions with different strategies. (unit: mm).

Solution	Annual Signal			Semi-Annual Signal		
	East	North	Up	East	North	Up
unit_xxx_s2	1.12	1.16	2.21	0.43	0.41	1.06
unit_tr_s2	1.15	1.17	2.21	0.42	0.42	1.05
unit_trs_s2	1.14	1.16	2.20	0.43	0.41	1.04
cova_tr_s2	1.14	1.14	2.19	0.35	0.38	0.96
cova_tr_s2d2	1.56	1.44	3.03	0.42	0.45	1.10
